# Long-Term Stability and Osteogenic Activity of Recycled Polysulfone-Calcium Silicate Bone Implants In Vitro

**DOI:** 10.3390/jfb16010031

**Published:** 2025-01-17

**Authors:** Chi-Nan Chang, Yun-Ru Huang, Shinn-Jyh Ding

**Affiliations:** 1 Institute of Oral Science, Chung Shan Medical University, Taichung City 402, Taiwan; danycnc@gmail.com (C.-N.C.); iris50887@gmail.com (Y.-R.H.); 2 School of Dentistry, Chung Shan Medical University, Taichung City 402, Taiwan; 3 Department of Stomatology, Chung Shan Medical University Hospital, Taichung City 402, Taiwan

**Keywords:** polysulfone, calcium silicate, osteogenesis, biostability, bone implant

## Abstract

Environmental protection issues have received widespread attention, making material recycling increasingly important. The upcycling of polysulfone (PSF) medical waste, recognized as a high-performance plastic with excellent mechanical properties, deserves promotion. While PSF is suitable for use as an orthopedic implant material, such as internal fixation, its osteogenesis capabilities must be enhanced. Mechanical stability, particularly over the long term, is a significant concern for bone implants in load-bearing applications. This study recycled PSF medical waste to create bone composites by incorporating osteogenic calcium silicate (CaSi) at three different contents: 10%, 20%, and 30%. We evaluated the phase, morphology, weight loss, and three-point bending strength of the PSF-based composites after they were soaked in dynamic simulated body fluid (SBF) at pH levels of 7.4 and 5.0 for up to 12 months. Human mesenchymal stem cells (hMSCs) were utilized to assess the osteogenic activity of these composites. Our findings revealed that, while the bending strength of PSF-based composites declined with prolonged exposure to SBF, the dissolution of CaSi particles led to a manageable weight loss of about 4% after 12 months, regardless of pH 7.4 or 5.0. Importantly, the incorporation of CaSi into the PSF matrix exhibited a positive effect on the attachment and proliferation of hMSCs. The levels of alkaline phosphatase (ALP) and calcium deposits directly correlated with the CaSi content, indicating superior osteogenic activity. Considering biostability and osteogenic ability, the 20% CaSi-PSF composite demonstrated promise as a candidate for load-bearing implant applications.

## 1. Introduction

The widespread use of plastics around the globe, coupled with low recycling rates, has substantially increased plastic waste, posing a major threat to our environment. To combat this issue, reducing, reusing, and recycling principles—known as the “3Rs”—are critical for promoting sustainability. The recycled use of polymers has emerged as a compelling solution to mitigate environmental impact [1,2,3,4]. Polysulfone (PSF), a high-performance engineering plastic, stands out as the premier biomaterial for applications in contact with blood thanks to its chemical inertness, thermodynamic stability, and exceptional biocompatibility [5,6,7]. Its remarkable mechanical properties, including strength and modulus, make PSF an ideal candidate for load-bearing hard-tissue prostheses [4,8,9]. The biomaterials for load-bearing implants are designed to have adequate mechanical properties, ensuring proper functionality during the bone repair process [10]. In light of these advantages and the pressing need for sustainable practices, exploring the recycling of PSF fibers from dialysis tube waste offers a valuable opportunity for creating eco-friendly polymer-based materials that can significantly contribute to reducing plastic waste and promoting a greener future.

There is an urgent clinical need to develop non-metallic implant materials that minimize the stress shielding effect, eliminate the requirement for follow-up surgeries to remove metallic components, and prevent metal artifacts that can complicate postoperative diagnostic imaging. High-strength polymers are a viable solution due to their lower modulus and density than metallic counterparts, making them suitable for bone implants and an attractive alternative to metals [4,8,11,12]. Moreover, flexible devices effectively stimulate the formation and development of calluses, thus enhancing the process of bone healing [13,14]. However, polymer matrix composites must exhibit osteogenic properties to ensure rapid bone healing in load-bearing conditions. Research by Bhat et al. demonstrated that incorporating hydroxyapatite (HA) into polymethyl methacrylate/PSF composites can improve biocompatibility for orthopedic prosthetic applications [15]. Meanwhile, Chang’s research team also innovated by creating bone plates from glass/polypropylene fabric composites, conducting in-depth studies on bone healing dynamics, and designing internal fixation devices, including bone plates [16].

The burgeoning use of PSF in load-bearing biomaterial is somewhat disconnected from clinical requirements, particularly in osteogenesis. Bioactive compounds can be incorporated into the PSF bulk or attached to its surface to enhance its osteogenic properties. This modification can create an active reaction area that promotes the formation of new bone tissue. Calcium silicates (CaSi) have garnered significant interest owing to their excellent osteogenic potential [17,18,19]. Our recent work has demonstrated that PSF-based composites could serve as effective implant materials, leveraging the mechanical properties of PSF alongside the biological properties of CaSi bioceramics [4]. Nonetheless, the long-term mechanical stability of these materials remains critical for clinical applications [12,20]. Thus, this study aimed to evaluate the long-term stability of the mechanical properties of the PSF/CaS composites through in vitro exposure to dynamic SBF at pH levels of 7.4 and 5.0 for up to 12 months. Moreover, using hMSCs provided valuable insights into the osteogenic activity of these composites, highlighting their potential for biomedical applications. These composites were developed using three different types of PSF (fiber and particle sourced from medical waste and particle from a commercial product) alongside varying ratios of CaSi fillers.

## 2. Materials and Methods

### 2.1. Preparation of PSF

The long hollow fiber PSF medical waste in the FX80 classix dialyzer (Fresenius Medical Care AG, Bad Homburg, Germany) was donated from the Green Plastic Technology Corporation (Tainan, Taiwan). The recycled PSF fibers were cut and sieved to facilitate the preparation of composites. Commercial PSF particles (Sigma-Aldrich, St. Louis, MO, USA) were used as controls to verify the performance of the recycled materials.

To prepare PSF particles, N-N-Dimethylacetamide (ECHO Chemicals, Miaoli, Taiwan) was used to dissolve either the Sigma-Aldrich PSF pellets or the recycled PSF fibers at 60 °C for one hour, after which the mixture was cooled in ice water [4]. Nanoparticles were produced through suction filtration and then dried at 60 °C. For convenience, samples of recycled fiber material were labeled RFB, while particles prepared from recycled fiber and commercial products were referred to as RNP and CNP, respectively.

### 2.2. Preparation of CaSi

The chemical precipitation method was used to prepare CaSi particles, as previously described [18]. In brief, calcium nitrate (Showa, Tokyo, Japan) was added to a cetyltrimethylammonium bromide (Sigma-Aldrich) solution containing 2% ammonia (Wako, Osaka, Japan). Subsequently, ethanol and tetraethyl orthosilicate (Sigma-Aldrich) were sequentially introduced to the mixture and stirred for 24 h. After centrifugation, the precipitates were washed with deionized water and dried in an oven overnight at 120 °C. They were then calcined in air at 800 °C for three hours to obtain the final particles.

### 2.3. Preparation of PSF-Based Composites

The PSF/CaSi composites were prepared by incorporating CaSi filler at different volume ratios (9:1, 8:2, and 7:3) into the PSF matrix using a conditioning mixer (ARE-250, Thinky, Tokyo, Japan) for 10 min. To aid in identification, the samples were labeled with the prefixes RFB, RNP, and CNP, accompanied by the volume ratio number. For instance, “RNP82” indicated a composite material that contained 20% CaSi particles mixed with recycled PSF particles. The green body was created by applying a pressure of 200 MPa for one minute.

Subsequently, a heat treatment was conducted at 240 °C for three hours, with a heating rate of 2 °C/minute in air. This step was essential for binding the PSF matrix with CaSi filler, after which the samples were cooled to room temperature.

### 2.4. In Vitro Stability

Dynamic SBF solution is an effective assay for simulating the continuous circulation of physiological fluids [20]. It was used to assess in vitro stability. The exchange process maintained constant ionic concentration and pH levels in the SBF by providing a fresh solution. Two SBF solutions with different pH values, 7.4 and 5.0, were prepared using hydrochloric acid and tris(hydroxymethyl)aminomethane for adjustment. The pH 5.0 was chosen to simulate the acidic environment associated with bacterial infections. To ensure consistent contact between all sample surfaces and the SBF volume, the surface-to-volume ratio was maintained at 0.1 cm^−1^. After soaking for specific durations (1, 3, 6, and 12 months), ten samples were taken from the vial to determine three-point bending strength. The remaining samples were dried in an oven at 60 °C to analyze weight loss, phase composition, and morphology.

### 2.5. Phase Composition and Surface Morphology

The impact of soaking time on the phase composition of various PSF composites was analyzed using a Bruker X-ray diffractometer (XRD; Karlsruhe, Germany) with Ni-filtered Cu Kα radiation at 40 kV and 40 mA. The scanning speed was set to 1° per minute. Additionally, a JEOL JSM-7800F field-emission scanning electron microscope (SEM; Tokyo, Japan) was employed to observe the changes in surface morphology of the materials after soaking.

### 2.6. Measurement of Weight Loss

Weight loss was measured using a four-digit balance (AE 240S, Mettler-Toledo AG, Greifensee, Switzerland). The dried samples were weighed until a constant weight was achieved before soaking (day 0) and after. Each group was examined with ten repeated samples at each time point.

### 2.7. Measurement of Three-Point Bending Strength

Three-point bending tests followed ASTM D790 standards [21] using rectangular bar specimens with 5 × 2 × 40 mm^3^ dimensions. The tests were conducted using an AGS-10kNX machine (Shimadzu, Kyoto, Japan) with a span of 32 mm. The three-point bending strength for each sample was obtained based on the formula σ = 3 PL/2 wh^2^, where P represents the peak load (N), L is the distance (mm) between the supports, w is the width (mm), and h is the height (mm) of the sample. The reported data for each composition corresponded to the mean of ten independent measurements.

### 2.8. In Vitro Osteogenic Activity

The in vitro osteogenic activity of the materials was evaluated using hMSCs obtained from Cell Engineering Technologies (Coralville, IA, USA). The samples, measuring 1 mm in thickness and 12 mm in diameter, were sterilized with 75% ethanol and exposed to ultraviolet (UV) light for two hours before cell incubation, using cells at passages 3–6. The hMSCs were then seeded at a density of 10^4^ cells per well on the sterilized samples in 48-well plates. The cell growth medium consisted of Dulbecco’s modified Eagle medium (DMEM; HMSC.E.Media-450, Cell Engineering Technologies), supplemented with 10% fetal bovine serum (FBS; Gibco, Langley, OK, USA) and 1% penicillin/streptomycin solution (Gibco). The cultures were maintained in an environment with 5% CO_2_ at 37 °C, with the medium changed every two days.

### 2.9. Cell Attachment and Proliferation

Cell attachment was assessed at 6 and 24 h, and cell proliferation was evaluated on days 3 and 7, which were examined using the MTT (3-(4,5-dimethylthiazol-2-yl)-2,5-diphenyltetrazolium bromide; Sigma-Aldrich) assay. Following a previously established protocol [19], the results were measured using a BioTek Epoch spectrophotometer (Winooski, VT, USA) at a wavelength of 563 nm. Three independent measurements were obtained to calculate the mean values.

### 2.10. ALP Activity and Mineralization

The ALP activity of hMSCs was examined at a cell density of 5 × 10^3^ cells per well after 7 and 14 days of incubation. The growth medium was replaced with an induction medium enriched with 10 nM of dexamethasone, 10 mM of β-glycerophosphate, and 0.05 mM of ascorbic acid to promote osteogenic differentiation. ALP activity was measured using an ALP assay kit (Takara, Shiga, Japan), and the detailed assay procedure was described elsewhere [20]. A total of three separate experiments were performed. To examine the synthesis of a mineralized matrix by hMSCs, Alizarin Red S staining was performed after 14 and 21 days of culture. The assay protocol was based on a previously established method [20]. The calcium mineral precipitate secreted by the hMSCs was destained using 10% cetylpyridinium chloride (Sigma-Aldrich) in PBS for 30 min to quantify matrix mineralization. Subsequently, the absorbance of the extract was measured using a BioTek Epoch spectrophotometer. Three replicates were conducted for each group.

### 2.11. Statistical Analysis

Statistical analysis was conducted using a one-way ANOVA, followed by Duncan’s multiple comparisons. A difference was considered statistically significant if *p* < 0.05.

## 3. Results

### 3.1. Phase Composition

**Figure 1:**
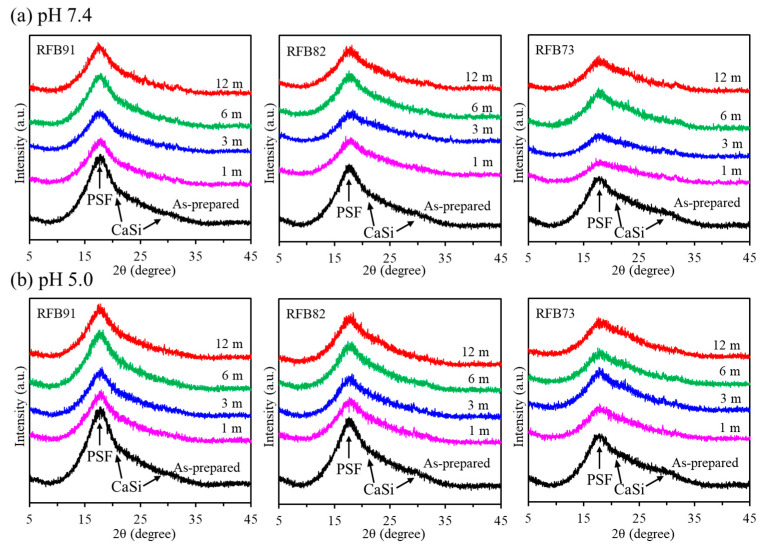
XRD patterns of three RFB composites with different CaSi content after soaking in an SBF solution at (**a**) pH 7.4 or (**b**) pH 5 for specified durations.

**Figure 2:**
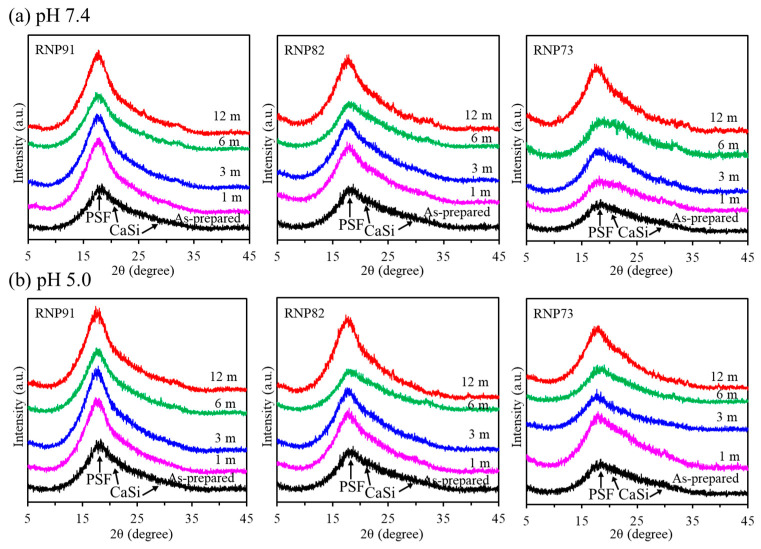
XRD patterns of three RNP composites with different CaSi content after soaking in an SBF solution at (**a**) pH 7.4 or (**b**) pH 5 for specified durations.

**Figure 3:**
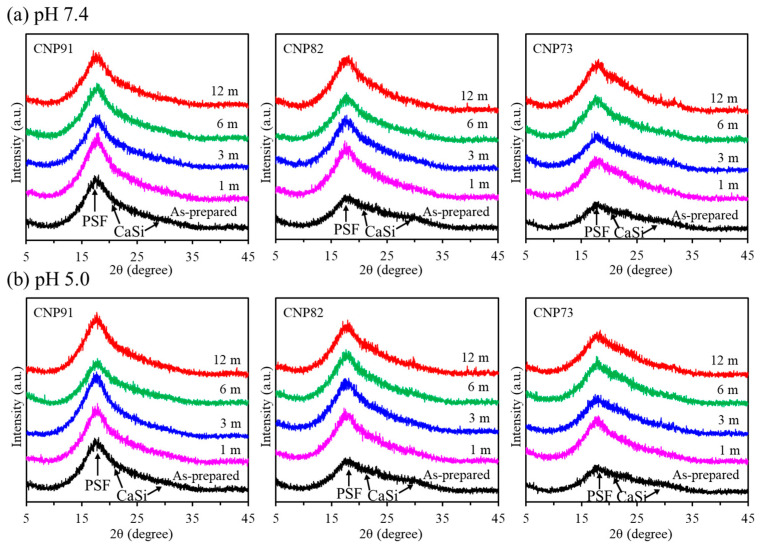
XRD patterns of three CNP composites with different CaSi content after soaking in an SBF solution at (**a**) pH 7.4 or (**b**) pH 5 for specified durations.

As a load-bearing composite, verifying the potential variations in material properties when immersed in a physiological solution for long-term stability is essential. We soaked the composite in two different pH SBF solutions (7.4 and 5.0) for up to 12 months to investigate this. Regardless of the CaSi contents—10, 20, or 30 vol%—the XRD patterns of the as-prepared composites displayed a broad peak at 2θ = 15–20°, attributed to the amorphous PSF phase [4]. At higher CaSi contents, broad and diffuse peaks in the 2θ = 20–30° range indicated the presence of amorphous SiO_2_, CaSiO_3_, and CaCO_3_ phases [4]. After soaking in a dynamic pH 7.4 SBF solution (Figure 1a), the XRD patterns of the three recycled PSF fiber-based composites did not show obvious changes over the soaking period. However, there was a slight decrease in the intensity of the CaSi peaks. The results for the pH 5.0 solution exhibited a similar trend to those observed in the pH 7.4 environment (Figure 1b). For the RNP-based composites (Figure 2), the phase composition of all composites remained unchanged throughout the 12-month soaking period. Additionally, the data illustrated in Figure 3 demonstrated that neither the solution pH nor soaking time significantly impacted the phase composition of PSF-based composites derived from the commercial product.

### 3.2. Morphology

**Figure 4:**
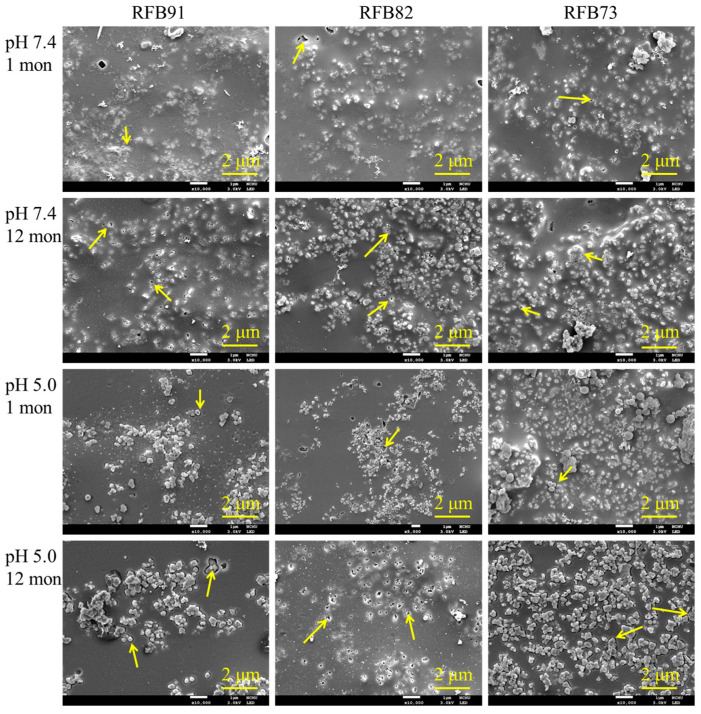
Surface SEM images of three RFB composites after being soaked in SBF solutions at pH levels of 7.4 and 5.0 for durations of 1 and 12 months. The scale bar represents two micrometers. Arrows indicate the solution-etched points surrounding the CaSi particle and the CaSi particles themselves.

The challenge of XRD in identifying small changes on masked surfaces can be addressed using the SEM technique. Broad-face SEM images of three recycled PSF fiber-based composites, which were soaked in SBF at pH levels of 7.4 and 5.0 for durations of 1 and 12 months, are displayed in Figure 4. The CaSi granular particles were integrated into the smooth structure of the PSF matrix. Notably, changes occurred on each surface during the soaking process in the SBF solution at both pH levels. After one month, it was evident that the CaSi particles were etched and dissolved, affecting the areas surrounding the CaSi and the particles themselves, as indicated by the arrows. Furthermore, the etching effect was intensified after 12 months of exposure. The environment at pH 5.0 had a slightly more pronounced impact than at pH 7.4.

**Figure 5:**
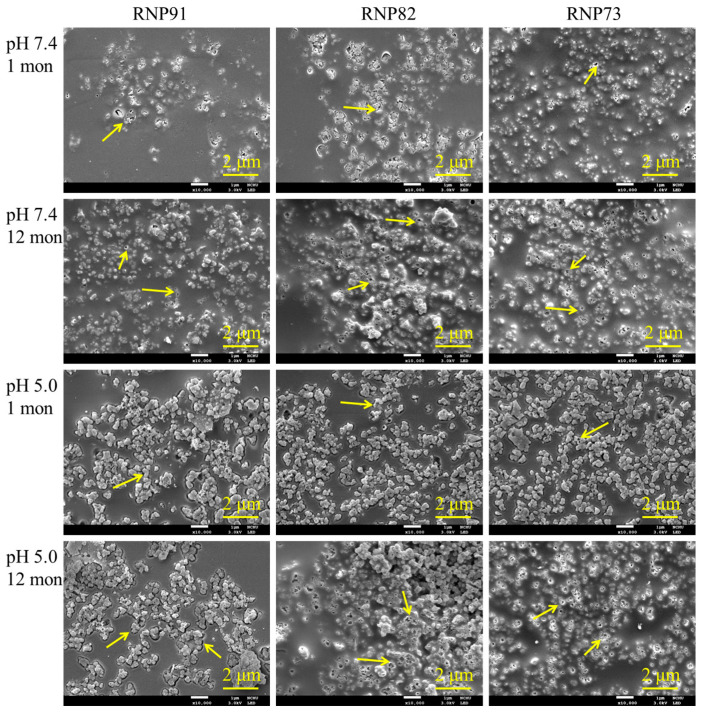
Surface SEM images of three RNP composites after being soaked in SBF solutions at pH levels of 7.4 and 5.0 for durations of 1 and 12 months. The scale bar represents two micrometers. Arrows indicate the solution-etched points surrounding the CaSi particle and the CaSi particles themselves.

**Figure 6:**
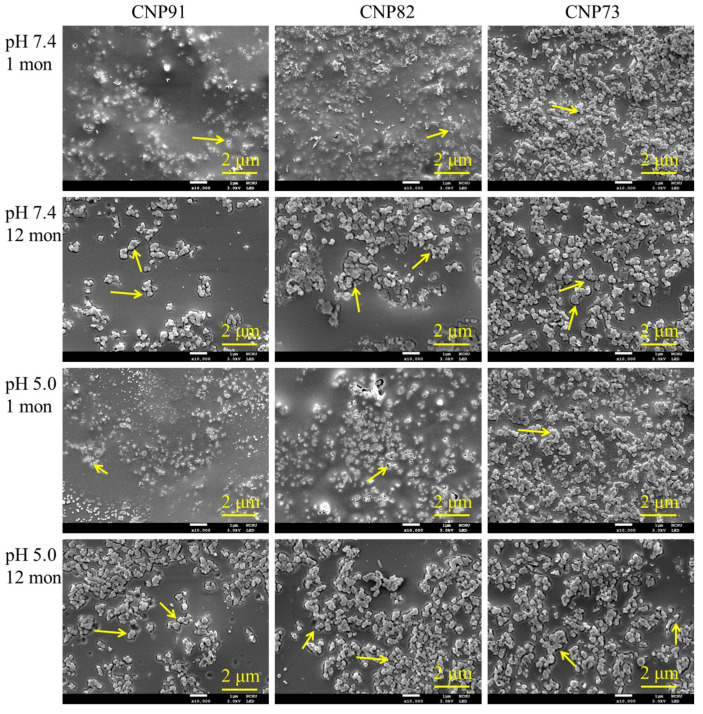
Surface SEM images of three CNP composites after being soaked in SBF solutions at pH levels of 7.4 and 5.0 for durations of 1 and 12 months. The scale bar represents two micrometers. Arrows indicate the solution-etched points surrounding the CaSi particle and the CaSi particles themselves.

Regardless of whether the samples were soaked in SBF with a 7.4 or 5.0 for one month, the surface morphology of the RNP group samples changed, showing the emergence of etching-induced nanopores. This was especially evident in samples with a high CaSi content (Figure 5). After being soaked for 12 months, the microstructures of these composites displayed profound surface etching and dissolution of the CaSi particles, with the arrows indicating the solution-etched points. A similar morphology was observed on the surfaces of CNP-based composites (Figure 6), which showed behavior identical to that of RFB-based composite materials during soaking. Both solution pH levels led to comparable etching phenomena.

### 3.3. Three-Point Bending Strength

**Figure 7:**
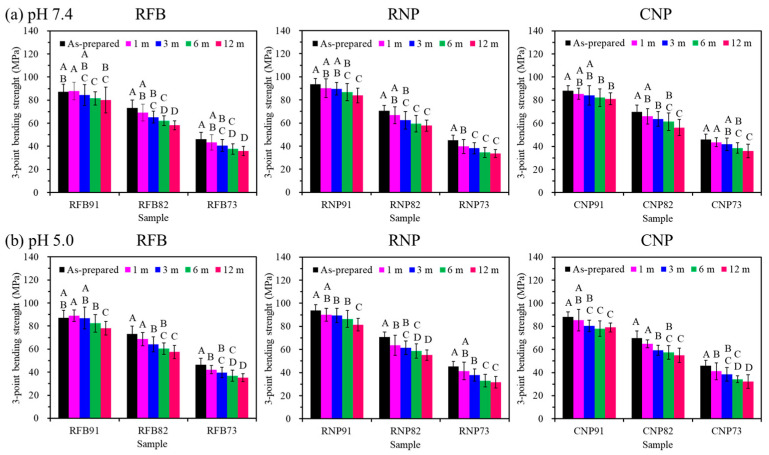
The three-point bending strength of various PSF-based composites with different CaSi contents before and after soaking in an SBF solution at two pH levels: (**a**) pH 7.4 and (**b**) 5.0 for predetermined durations. Statistical comparisons were made between the different soaking time points for the same composite. Different capital letters indicate significant differences, with a significance level of *p* < 0.05 (*n* = 10).

Figure 7 illustrates the changes in the three-point bending strength of three PSF-based composites before and after soaking in solutions with pH levels of 7.4 (a) or 5.0 (b) for up to 12 months. The results indicated that soaking time significantly affected (*p* < 0.05) the bending strength of all composites immersed in pH 7.4 and 5.0 solutions, with materials experiencing strength loss as soaking duration increased. After one month of soaking, strength had almost no decrease for any groups. In contrast, after three months of soaking, the bending strength of the composites with higher CaSi content (groups 82 and 73) showed a significant reduction (*p* < 0.05) compared to the corresponding as-prepared composites. Specifically, the RFB82 and RFB73 composites experienced a loss of 11% and 12% bending strength, respectively, after three months in a pH 7.4 solution. At the more acidic pH 5.0 environment, the strength loss was 12% and 14%, respectively. Furthermore, after six months of soaking in pH 5.0 SBF, the strength of the RFB91, RNP91, and CNP91 composites decreased by 5%, 8%, and 11%, respectively, compared to their original strength. Throughout all soaking periods, the bending strength values of group 82 were consistently higher than those of group 73, regardless of the PSF types. After soaking for 12 months in pH 7.4 SBF, the strength values of RFB82, RNP82, and CNP82 decreased by 20%, 18%, and 19%, respectively. In contrast, the strength reductions were 22%, 22%, and 23% in the pH 5.0 environment. Notably, the lower pH environment led to more obvious strength degradation of the composites. It is essential to highlight that, even after 12 months of soaking in either the pH 7.4 or 5.0 solution, the 82 groups maintained a three-point bending strength of over 55 MPa (Table 1), which falls within the reported range of flexural strength for cortical bone (50–150 MPa) [4].

### 3.4. Weight Loss

**Figure 8:**
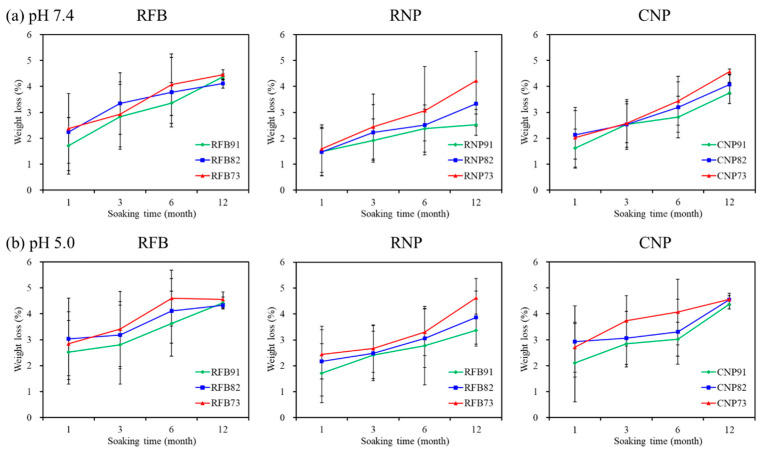
Changes in weight loss of various PSF-based composites with different CaSi contents before and after soaking in an SBF solution at (**a**) pH 7.4 or (**b**) pH 5.0 for extended periods (*n* = 10).

Figure 8 shows the weight loss of the composites after being exposed to solutions with pH 7.4 and 5.0 for up to 12 months. The weight loss showed a slight increase with longer soaking times. For the composites immersed in the pH 7.4 solution (Figure 8a), variations in CaSi content did not remarkably impact the weight loss. Similarly, the PSF-based composites revealed minimal weight loss of only 2–3% after one month of soaking in the acidic solution with a pH of 5.0 (Figure 8b). By the end of the 12-month soaking period, all composites experienced a consistent weight loss of approximately 4%, irrespective of the PSF type and CaSi content used. This finding demonstrated the long-term stability of these composites under different pH conditions.

### 3.5. Cell Attachment and Proliferation

**Figure 9:**
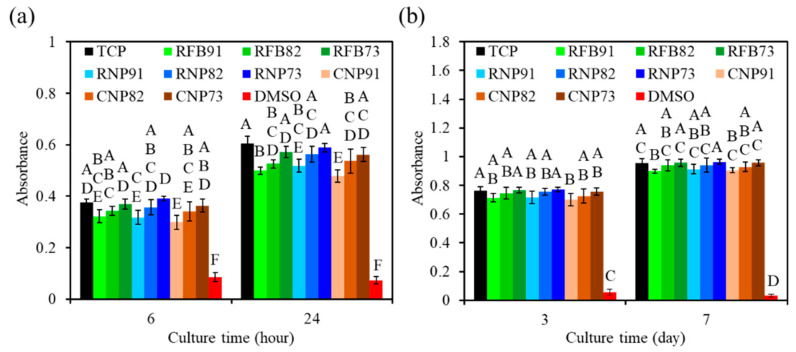
(**a**) Cell attachment and (**b**) proliferation of hMSCs cultured on various composite surfaces at different time points. Statistical comparisons were made between samples incubated for the same duration. Different capital letters indicate significant differences at *p* < 0.05 (*n* = 3).

The effect of CaSi content in three types of PSF-based composites on the in vitro osteogenic activities of hMSCs was studied. As shown in Figure 9, the absorbance, which indicates cell growth, increased for all composites from 6 h to 7 days of culture time. Moreover, irrespective of the type of PSF, higher CaSi content in the composites significantly enhanced (*p* < 0.05) cell growth at all measured culture time points. For example, after 6 h of culture, the cells on the RNP73 composite showed an MTT absorbance of 0.39, significantly higher (*p* < 0.05) than the 0.32 absorbance measured for the RNP91 composite. At 24 h of culture, hMSCs grown on the RFB73 composite displayed a 15% higher attachment than those on the RFB91 composite (Figure 9a). Regarding cell proliferation, all PSF-based composites, except for the DMSO group, achieved over 90% viability when normalized to the TCP control during the culture periods (Figure 9b). For example, on day 7, the TCP control had an absorbance of 0.96, while the CNP91, CNP82, and CNP73 composites measured 0.91, 0.93, and 0.96, respectively. Notably, the composites with identical CaSi content from different PSF types exhibited consistent cell proliferation, showing no significant differences (*p* > 0.05) throughout the culture periods.

### 3.6. ALP Activity and Mineralization

**Figure 10:**
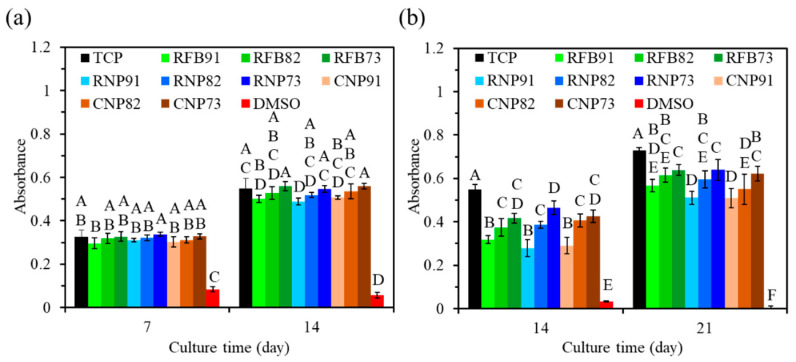
(**a**) The absorbance levels of (**a**) alkaline phosphatase activity and (**b**) mineralization expression in hMSCs cultured on various PSF-based composite surfaces. Samples that were incubated for the same duration were subjected to statistical analysis. Statistically significant differences are indicated using different capital letters, with a *p*-value of less than 0.05 (*n* = 3).

The ALP activity consistently indicated that the PSF-based composites with higher CaSi content exhibited greater ALP expression (Figure 10a). After 7 days of culture, the ALP expression of hMSCs on the composite materials showed no significant difference (*p* > 0.05). Notably, on day 14, the ALP levels of hMSCs cultured in the 73 groups, which included RFB, RNP, and CNP, increased significantly (*p* < 0.05) by 11%, 12%, and 10%, respectively, compared to the 91 groups. There were no significant differences (*p* > 0.05) between the 82 groups and the 73 groups. For example, compared to RNP 82 with an ALP level of 0.52, RNP 73 was 0.55. Moreover, after 14 days of culture, RFB82 exhibited ALP levels comparable to those of RNP82 and CNP82, with no significant difference observed (*p* > 0.05). Additionally, similar results were observed in the 91 and 73 groups. These findings demonstrated that the PSF types did not impact ALP expression, highlighting the consistency of ALP levels across different PSF types.

The hMSCs on the surfaces of the composites with higher CaSi content demonstrated higher levels of the mineralized matrix (Figure 10b). After 14 days of culture, significant differences (*p* < 0.05) were observed between the composites. The calcium deposit values for RFB 91, RFB 82, and RFB 73 were 0.32, 0.37, and 0.42, respectively. Likewise, RNP 91, RNP 82, and RNP 73 had mineralization values of 0.28, 0.39, and 0.46, respectively. By day 21, a remarkable enhancement of approximately 20% in mineralized content was detected in the 73 groups compared to the 91 groups. For example, the mineralization value of hMSCs on the CNP91 composite was 0.51, significantly lower (*p* < 0.05) than the 0.62 value for the CNP73 composite. In comparing recycled PSF (RFB and RNP) with virgin PSF (CNP), both containing equivalent CaSi levels, it is noteworthy that no significant differences were found in mineralization capability (*p* > 0.05).

## 4. Discussion

Upcycling PSF waste materials is appealing from ecological and economic perspectives [3,4]. Good osteogenesis and mechanical compatibility are essential for load-bearing implants, as these factors promote effective bone ingrowth and create a strong bond between the bone tissue and the implants. This can enhance the stability of the implants and facilitate the formation of new bone during the early stages of healing. More importantly, the long-term stability of the biomaterial is essential for achieving the desired level of clinical success, specifically for older patients. This study selected bioactive CaSi as a reinforcement to enhance the osteogenesis in the composites. Research on osteogenesis and long-term in vitro studies was conducted to verify the advantages of recycled PSF waste plastics in developing new medical materials.

Implementing a simple in vitro model under dynamic conditions is essential for evaluating the long-term stability of implant materials. Local metabolic acidosis, driven by bacteria and osteoclasts, dramatically alters pH levels in bone lesions, reducing them from the normal level of 7.4 to an acidic 5.0 [20,22,23,24]. This shift has crucial implications for the behavior of implant materials, as their stability during soaking time is influenced by both the surrounding physiological environment (such as pH) and the materials’ properties (like solubility) [24,25]. By understanding these factors, we can more reliably predict the in vivo resorption behavior of these materials, thereby improving the reliability of preclinical trials. To address this need, a comprehensive study was conducted on the stability of PSF-based composites in a dynamic SBF with pH levels of 7.4 and 5.0 over 12 months. This study allowed for a thorough analysis of the long-term changes in the properties of the composites for practical applications.

**Scheme 1:**
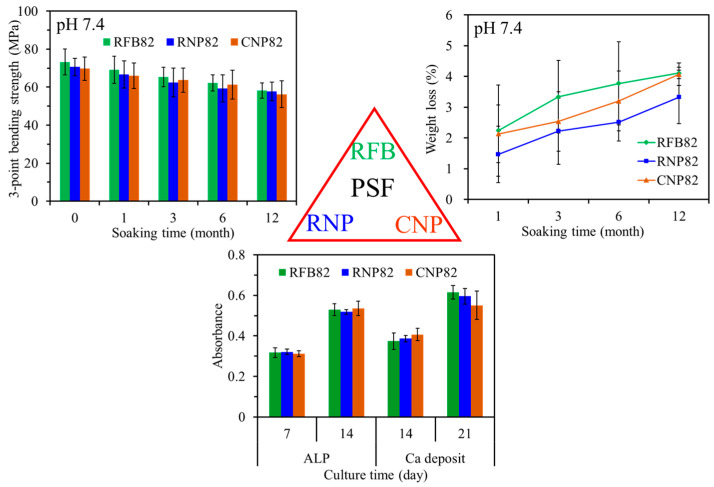
A schematic diagram for understanding the impact of PSF types with 82 groups on mechanical properties, weight loss, and osteogenic activity.

The results of the XRD analysis displayed that neither the solution pH nor the soaking time significantly impacted the phase composition of the three distinct PSF-based composites. However, SEM observation provided compelling evidence that the CaSi particles underwent etching, forming voids and crevices, the extent of which was related to soaking time and solution pH levels. At the same time, a decline in the mechanical properties of the composites during the soaking occurred, particularly in the group with a higher CaSi content. Mechanical stability is essential for bone implants, as it reflects the ability to maintain original mechanical properties without degradation over time, which is critical for the short- and long-term clinical success of load-bearing applications [12,20,26]. When bioactive CaSi was incorporated into a composite, it was essential to consider how this material affected the mechanical properties, especially when exposed to body fluids. The bending strength of the PSF-based composites decreased after immersion at pH levels of 7.4 and 5.0 with the increase in the soaking time, with the reduction being more pronounced at the lower pH. Brown et al. reported a 15% loss in flexural strength for carbon fiber-reinforced PSF composites following three weeks in saline [27]. Similarly, Meyer et al. found that interfacial bond strengths of carbon fiber-reinforced PSF diminished significantly when subjected to physiologic saline, influenced by time and temperature [28]. Ekstrand et al. noted that a substantial reduction in mechanical properties of the carbon fiber-reinforced polymethylmethacrylate was linked to hydrolytic degradation of the interfacial bonds [29]. In contrast to the soaking time effect, all PSF types (RFB, RNP, and CNP) with identical CaSi content demonstrated consistent mechanical strength (Scheme 1). Yu et al. evaluated the mechanical properties and long-term durability of virgin and recycled PSF plastics made from discarded PSF nonwovens, concluding that both types exhibited similar tensile properties [1]. This consistency underscored the potential of these materials in applications.

The decline in strength can be attributed to two significant mechanisms: the inadequate interaction between the PSF matrix and CaSi fillers and the solubility of the CaSi particles. The lack of bonding at the matrix–filler interface, as observed in the SEM images, suggests that water molecules and ions in the SBF may penetrate and disrupt the interfacial bond, potentially leading to debonding. Furthermore, the etched areas can serve as critical fracture initiation points during bending, ultimately contributing to structural failure [12]. Another contributing factor to the decreased strength was the solubility of the CaSi particles, as evidenced by weight loss measurements. Remarkably, the variations in weight loss were unaffected by the differences in PSF types (Scheme 1). Unlike the PSF matrix, the CaSi particles exhibited chemical instability within the composite, which led to surface reactions with external fluids. Throughout soaking, the weight of the PSF/CaSi composites reduced by approximately 4% after 12 months in a dynamic solution with pH levels of either 7.4 or 5.0, illustrating a minimal level of biodegradation in the surrounding environment. According to Oréfice et al., water migration through the interface between bioactive glass particles and the PSF matrix caused the surface dissolution of glass particles, forming voids around particles, which was responsible for the observed decline in mechanical properties [9]. To address these challenges and enhance interfacial stability, further research is crucial. Exploring binding agents, such as silanes, has shown the potential to significantly improve the bonding of composite materials, thereby enhancing the mechanical integrity of PSF-based composites. Strengthening this interface is critical to unlocking the full potential of these materials.

It is important to note that, although the soaking time reduced the three-point bending strength of PSF-based composites, those containing 20% CaSi from the three types of PSF still exhibited a strength of over 55 MPa after 12 months. This level of strength is well within the bending strength range of cortical bone (50–150 MPa) [4]. Such robust PSF-based composites could provide essential stability for bone union during the critical initial phase of fracture healing [12]. Still, they also possessed a relatively slow degradation vital for specific clinical applications like vertebroplasty [30]. Notably, the surface dissolution of bioactive CaSi ceramic plays a significant role in the osteogenic activity of these composites; the release of Ca and Si ions is likely to boost cell functions and stimulate bone tissue growth [31], as elaborated below.

One of the most important properties of bone implants is their ability to form strong bonds with surrounding bone tissue during the healing process. This can be achieved by using bioactive components that enhance the surface characteristics of the implant [17,20,32]. While a pure PSF polymer demonstrates good mechanical compatibility as an orthopedic implant material, such as for internal fixation, it requires reinforcement to promote osteogenesis, an essential process for harnessing the natural regenerative capabilities of bone [33]. Incorporating bioactive CaSi ceramic particles into PSF polymers altered the chemistry of the composite surface, facilitating better bonding to bone. To assess the significant effects of CaSi on the osteogenic activity of PSF-based composites, we evaluated the attachment, proliferation, differentiation, and mineralization of hMSCs cultured on these innovative composites. The findings demonstrated that introducing bioactive CaSi to PSF enhanced cellular responses, leading to the dose-dependent osteogenic activity of the composites. Moreover, studies have shown that CaSi bioceramics effectively promote hMSC differentiation and support bone formation [17,18,34]. The bioactive CaSi component further enhances implant stability by increasing the contact area, thereby reinforcing the anchorage of the implant in regions characterized by poor bone quality. The gradual release of bioactive CaSi was beneficial for promoting cell growth within the pores, which could create an interlocking effect with adjacent normal bone tissue, significantly enhancing the fixation of bone implants. Therefore, the incorporation of CaSi fillers into PSF not only improved osteogenesis in vitro but also held great promise for clinical applications. A previous study confirmed that CaSi demonstrated superior osteogenesis in a mini-pig model with a mandibular alveolar bone defect [17]. It is anticipated that the high osteogenic properties of these partially dissolvable PSF-based composites could shorten healing times, particularly for elderly patients. Non-surprisingly, non-degradable polymer matrices, such as high-density polyethylene (HDPE) [35,36] and polyetheretherketone (PEEK) [37] with bioactive fillers have been developed for bone replacement. For example, Bello et al. incorporated snail shells and pumpkin pod nanoparticles into HDPE to replace the trabecular bones in the thoracic lumbar region [36]. Zheng et al. found improved bonding strength between the HA/PEEK composite and host due to bioactive HA [37]. It is intriguing to note that variations among different types of PSF did not affect hMSC functions, emphasizing the crucial role of material composition (Scheme 1).

In summary, the composites derived from recycled PSF with fiber (RFB) or nanoparticles (RNPs) demonstrated properties equivalent to those of the virgin type (CNP). They showcased comparable mechanical strength, weight loss, and osteogenic activity of hMSCs, as illustrated in Scheme 1. This highlighted the potential of using recycled materials without compromising quality. It is important to note that the risk of non-union fractures in older adults dramatically increases due to osteoporosis and age-related changes in bone density [38]. D’Ippolito et al. highlighted a significant decline in the osteogenic potential of hMSCs due to aging [39]. This age-related deterioration in function is concerning, as it stems from reduced cell proliferation, impaired cell differentiation, and dysfunctional osteoprogenitor cells [40]. Tackling this challenge is vital for enhancing recovery outcomes and ensuring a better quality of life for seniors. This study demonstrated that, while the cellular responses of low-degradable PSF/CaSi composite implants increased with higher CaSi content, the overall enhancement in cell growth and osteogenic potential is not substantial. Therefore, applying a bioactive coating to these composites is essential to significantly improve their cell growth and osteogenic activity. Moreover, long-term in vivo studies are necessary to validate the bond between the bone tissue and the composite material, confirming the composites’ suitability for long-term load-bearing applications. This validation was essential, as the non-degradable PSF matrix played a significant role in the composite’s effectiveness. Finally, when considering treatment options for elderly patients, lightweight polymer-based composites stand out as a superior choice compared to heavy metallic implants. By minimizing weight-related stress, these innovative materials improve comfort and mobility, making them an excellent alternative for the aging population [41,42].

## 5. Conclusions

An innovative composite of recycled stable PSF plastics and bioactive CaSi bioceramics was developed for load-bearing implant applications, emphasizing sustainability. The results of this study indicated that, while incorporating CaSi filler did decrease the three-point bending strength of PSF-based composites when soaked in SBF at pH levels of 7.4 or 5.0, the PSF-based composites with 20% CaSi content from all three types of PSF still exhibited a bending strength exceeding 55 MPa even after 12 months. Moreover, a higher CaSi content in these composites led to increased levels of cell growth, ALP expression, and the development of a mineralized matrix in hMSCs. This study will pave the way for creating high-osteogenic implants with long-term stability that fulfill the reconstruction requirement for bone implants such as artificial vertebrae, intervertebral disks, dental implants, and bone fracture fixation devices. To this end, a silane binding layer may address the interface challenges between the PSF matrix and the CaSi filler. Additionally, an injection molding process could facilitate the scale-up production of these composites, while 3D printing technology may be employed in the custom design of the implants. To ensure these materials live up to their promises, an additional bioactive coating deposition and in vivo research are essential to evaluate their osteogenic potential and confirm their clinical benefits.

## Figures and Tables

**Table 1:** Three-point bending strength of various PSF-based composites before and after soaking in SBF with pH levels of 7.4 or 5.0.

Soaking Time (Month)		RFB			RNP			CNP	
	91	82	73	91	82	73	91	82	73
pH 7.4									
0	87.1 ± 6.5	73.2 ± 6.8	46.4 ± 5.8	93.1 ± 4.9	70.6 ± 4.6	45.2 ± 4.4	88.2 ± 4.3	69.8 ± 6.1	46.0 ± 4.6
1	87.8 ± 7.5	69.1 ± 7.2	43.3 ± 6.7	90.1 ± 8.2	66.7 ± 7.2	39.8 ± 6.1	85.5 ± 4.9	66.0 ± 6.7	43.8 ± 3.9
3	84.5 ± 9.1	65.3 ± 5.2	40.1 ± 5.1	89.7 ± 5.5	62.4 ± 7.6	38.2 ± 4.9	85.1 ± 8.4	63.7 ± 6.4	41.7 ± 5.4
6	81.5 ± 5.7	62.2 ± 4.3	37.7 ± 4.6	86.8 ± 7.4	59.4 ± 7.2	34.5 ± 4.4	82.2 ± 7.6	61.3 ± 7.5	38.6 ± 4.6
12	80.0 ± 11.2	58.2 ± 3.9	36.0 ± 4.0	83.8 ± 6.3	57.8 ± 4.8	33.6 ± 3.4	81.1 ± 5.1	56.2 ± 7.0	36.0 ± 5.8
pH 5.0									
0	87.1 ± 6.5	73.2 ± 6.8	46.4 ± 5.8	93.1 ± 4.9	70.6 ± 4.6	45.2 ± 4.4	88.2 ± 4.26	69.8 ± 6.1	46.0 ± 4.6
1	88.9 ± 5.1	68.7 ± 5.8	42.2 ± 3.5	90.1 ± 5.5	63.7 ± 8.7	41.4 ± 7.6	85.3 ± 9.4	64.9 ± 3.3	41.2 ± 7.3
3	86.9 ± 9.5	64.1 ± 6.4	36.9 ± 4.6	39.3 ± 6.3	61.4 ± 5.9	37.9 ± 5.3	80.4 ± 5.1	59.4 ± 4.4	38.4 ± 5.9
6	82.4 ± 7.6	60.3 ± 5.0	36.9 ± 4.9	86.5 ± 7.4	58.7 ± 6.3	33.0 ± 5.5	78.1 ± 6.8	57.4 ± 6.0	34.2 ± 2.8
12	78.1 ± 5.8	57.4 ± 5.8	35.2 ± 3.4	81.5 ± 5.4	55.1 ± 4.6	31.7 ± 4.9	79.0 ± 3.9	55.0 ± 6.2	32.3 ± 5.9

## Data Availability

The original contributions presented in this study are included in this article; further inquiries can be directed to the corresponding author.
